# Npn-1 Contributes to Axon-Axon Interactions That Differentially Control Sensory and Motor Innervation of the Limb

**DOI:** 10.1371/journal.pbio.1001020

**Published:** 2011-02-22

**Authors:** Rosa-Eva Huettl, Heidi Soellner, Elisa Bianchi, Bennett G. Novitch, Andrea B. Huber

**Affiliations:** 1 Institute of Developmental Genetics, Helmholtz Zentrum München–German Research Center for Environmental Health, Neuherberg, Germany; 2 Department of Neurobiology, Broad Center of Regenerative Medicine and Stem Cell Research, David Geffen School of Medicine at UCLA, Los Angeles, California, United States of America; University of Cambridge, United Kingdom

## Abstract

The initiation, execution, and completion of complex locomotor behaviors are depending on precisely integrated neural circuitries consisting of motor pathways that activate muscles in the extremities and sensory afferents that deliver feedback to motoneurons. These projections form in tight temporal and spatial vicinities during development, yet the molecular mechanisms and cues coordinating these processes are not well understood. Using cell-type specific ablation of the axon guidance receptor Neuropilin-1 (Npn-1) in spinal motoneurons or in sensory neurons in the dorsal root ganglia (DRG), we have explored the contribution of this signaling pathway to correct innervation of the limb. We show that Npn-1 controls the fasciculation of both projections and mediates inter-axonal communication. Removal of Npn-1 from sensory neurons results in defasciculation of sensory axons and, surprisingly, also of motor axons. In addition, the tight coupling between these two heterotypic axonal populations is lifted with sensory fibers now leading the spinal nerve projection. These findings are corroborated by partial genetic elimination of sensory neurons, which causes defasciculation of motor projections to the limb. Deletion of Npn-1 from motoneurons leads to severe defasciculation of motor axons in the distal limb and dorsal-ventral pathfinding errors, while outgrowth and fasciculation of sensory trajectories into the limb remain unaffected. Genetic elimination of motoneurons, however, revealed that sensory axons need only minimal scaffolding by motor axons to establish their projections in the distal limb. Thus, motor and sensory axons are mutually dependent on each other for the generation of their trajectories and interact in part through Npn-1-mediated fasciculation before and within the plexus region of the limbs.

## Author Summary

During embryonic development, growing axons establish intricate neural networks with their peripheral targets, a process that builds the basis for complex behaviors. While wiring up the proper circuits in peripheral limbs, for example, motor axons from the spinal cord and sensory axons from the dorsal root ganglia converge in the spinal nerve. Here, they intermingle and are subsequently sorted before reaching the plexus region, the pivotal dorsal-ventral choice point on their path to the limb. In this study, we analyzed the contribution of the axon guidance receptor Neuropilin-1 (Npn-1) to determine how axons choose their path, how well they are able to maintain their correct path, and how it influences the interactions between spinal sensory axons and motor axons. We find that when Npn-1 is eliminated from sensory neurons, both sensory and motor axons are “derailed” from their correct nerve bundles, and there is a break in the tight coupling between these axonal populations. Loss of Npn-1 in motoneurons, however, leads to impairments in axon bundling and pathfinding errors only in motor axons, while sensory axons remain unaffected. Genetic ablation studies of either sensory or motor neurons corroborate the results on the mutual dependency and specificity of the outgrowing spinal projections. These results reveal a role for Npn-1 in controlling specific axon-axon interactions that lead to formation of proper spinal sensory-motor trajectories to the limb. Furthermore, they suggest that the presence of minimal numbers of sensory or motor axons is sufficient for the formation of correct spinal projections.

## Introduction

The establishment of concerted locomotor behaviors in vertebrates relies on the formation of integrated motor and sensory circuits that form between defined populations of neurons and their appropriate targets in the periphery. During the development of the spinal neuromuscular circuitry, motoneurons and sensory neurons of the dorsal root ganglia (DRG) align their axons to form spinal nerves that extend together over long distances towards their respective peripheral targets in the developing limb. The molecular mechanisms underlying the early organization of these projections, from diverse origins into tight fascicles, and subsequent sorting into target-specific bundles are crucial to their pathfinding success, but are not well understood. Several families of complementary receptor-ligand pairs that are expressed on projecting neurons and their targets and serve as axon guidance cues have been identified over the last two decades [1]–[3]. The expression of these secreted and membrane-bound factors, and their neuronal receptors, is tightly regulated, both spatially and temporally.

However, growing axons recognize and respond to cues presented not only in intermediary and final target tissues but also on neighboring axons. In the fly olfactory system, axon-axon interactions mediated by Sema1A expressed on early-arriving axons to the antenna constrain the choice of late-arriving axons from the maxillary palp, likely through repulsive interactions with PlexinA, a receptor for Sema1A [4]. Recently, the same family of axon guidance molecules has been implicated in pre-target segregation of axons that project to different regions of the mouse olfactory bulb. Genetic experiments revealed that these pre-target axon-axon interactions are mediated by expression of complementary amounts of Neuropilin-1 (Npn-1) and Semaphorin 3A (Sema3A) on olfactory sensory neurons and result in axonal segregation en route to their target destinations, specific and unique glomeruli in the olfactory bulb [5].

During the development of the vertebrate limb, axon-environment interactions mediated by molecules of several axon guidance cue families play crucial roles in the establishment of precise connectivity in sensory-motor circuitry. Motoneurons that innervate limb muscles reside at brachial and lumbar levels in the ventral spinal cord and form the lateral motor columns (LMC). Lateral LMC neurons (LMCl) express EphA4 and are guided to the dorsal limb through repulsive interactions with ephrinA ligands [6]–[9] while repulsive Sema3F-Npn-2 as well as ephrinB-EphB signaling directs axons from the medial LMC (LMCm) to ventral limb muscles [10],[11]. Selective fasciculation and de-fasciculation of sensory and motor nerves within specific decision areas, e.g. the plexus region, are also controlled by axon-environment interactions [10],[12],[13]. Only recently inter-axonal signals between co-extending sensory and motor axons have been proposed to organize these projections within nerves innervating axial muscles of the trunk. Indeed, repulsive interactions mediated through ephrinA ligands expressed on sensory axons and EphA3/A4 receptors present on motoneurons of the medial aspect of the medial motor column (MMCm) result in a sharp pre-target segregation of motor and sensory pathways [14]. However, the role of axon-axon interactions in the formation of non-axial projections, for example in the trajectories to the limb, remains unclear. Deletion of motoneurons through surgical removal of several segments of neural tube in the developing chick suggested that motor axons influence the patterning of sensory trajectories either by axons providing environmental and/or selective fasciculation cues to guide sensory axons [15]. Subsequent studies showed that sensory axons retain the capability of finding their correct targets if motoneurons are removed after the developmental stage when neural crest cells coalesce into the DRG. This suggests that the sensory neuron identity or axon extension is plastic with regard to pathway and target choice [16]. While these data show a dependence on correct motor axon growth for correct sensory axon outgrowth and guidance, they do not rule out the converse.

Class 3 semaphorin mediated signals govern several distinct aspects of the formation of spinal sensory-motor connectivity: in contrast to repulsive Sema3F-Npn-2 interactions, which guide a subset of medial LMC axons into the ventral forelimb, Sema3A-Npn-1 signaling directs the fasciculation, timing, and fidelity of motor axon growth into the forelimb [10]. Interestingly, Npn-1 is expressed not only at early time points in all LMC neurons projecting to the limb, but also in sensory neurons of the DRG, and is therefore in a position to mediate axon-axon interactions between sensory and motor fibers during development.

In this study, using genetic tools, we re-examined the reciprocal interactions of sensory and motor axons as they navigate their trajectories and explored the role of Npn-1 signaling in the communication between these two peripheral nerve components. We show that Npn-1 is required in sensory axons to maintain proper fasciculation and organization of both sensory and motor axons. DRG-specific removal of Npn-1 leads to defasciculation of motor projections even though Npn-1 is still present in motoneurons. In line with these findings, peripheral motor projections can still form if sensory neurons are partially eliminated by activation of diphteria toxin fragment A (DT-A), however motor trajectories become defasciculated. Elimination of Npn-1 from motoneurons, however, leads to defasciculation of motor projections beyond the plexus without influencing the correct formation of sensory trajectories. This defasciculation of motor axons also resulted in dorsal-ventral guidance errors within the limb. Partial elimination of motoneurons resulted in markedly thinned, or even absent, sensory projections depending on the degree of motoneuron reduction. Our results underscore the crucial role of Npn-1 signaling for the sorting and selective fasciculation of sensory and motor axons of the vertebrate limb prior to these projections arriving at the important early choice point, the plexus region. Our data show that correct fasciculation and the presence of either motor or sensory axons proximal to the plexus region play a key role in the development of both classes of projections.

## Results

### Fasciculation of Motor Axons Is Controlled by Npn-1 Expressed in Motoneurons

**Figure 1:**
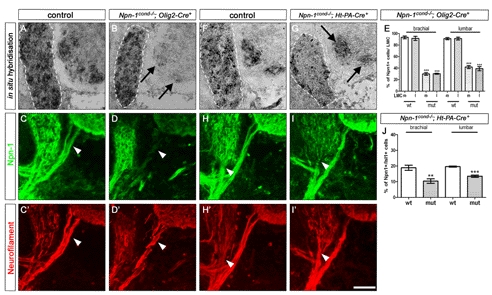
Quantification of Cre recombinase efficiency. In situ hybridization against the floxed exon 2 of *Npn-1* demonstrates ablation of *Npn-1* from motor neurons (arrows in B) but not from DRG (outlined with a white dashed line) in *Npn-1^cond^* ^−*/*−^ *;Olig2-Cre^+^* mutant embryos; littermate control in (A). Quantification (E) reveals that in control embryos nearly 100% of medial and lateral LMC neurons (positive for Isl-1 and Lim-1, respectively) at brachial and lumbar levels express *Npn-1*, whereas in mutant embryos a decrease to 29.8%±2.3% in medial, 30.1%±0.8% in lateral (*p*<0.0001) brachial neurons, and 41.8%±2.6% in medial and 39%±3.6% in lateral (*p*<0.0005) lumbar neurons was observed. Absence of Npn-1 protein is visualized by immunohistochemistry against Npn-1. In *Npn-1^cond^* ^−*/*−^ *;Olig2-Cre^+^* mutant embryos Npn-1 is absent from motor nerve braches (arrowhead in D, wildtype littermate in C), whereas Npn-1 expression is not affected in sensory trajectories. In situ hybridization on *Npn-1^cond^* ^−*/*−^ *;Ht-PA-Cre^+^* mutant embryos (G, littermate control in F) reveals ablation of *Npn-1* selectively from DRG, but not from motor neurons (arrows in D). Quantification (J) shows a 2-fold decrease in the numbers of *Npn-1* expressing sensory neurons (positive for Isl-1) in mutant embryos to 10.4%±0.8% at brachial and 13.5%±0.4% at lumbar levels (*n* = 3, *p* ^brachial^<0.005; *p* ^lumbar^<0.0005). In *Npn-1^cond^* ^−*/*−^ *;Ht-PA-Cre^+^* mutant embryos Npn-1 protein expression is unchanged in motor projections, whereas its presence in sensory fibers is markedly reduced (arrowhead in I) when compared to littermate controls (H). Bar equals 50 µm in all panels (*n* = 3 for all genotypes).

**Figure 2:**
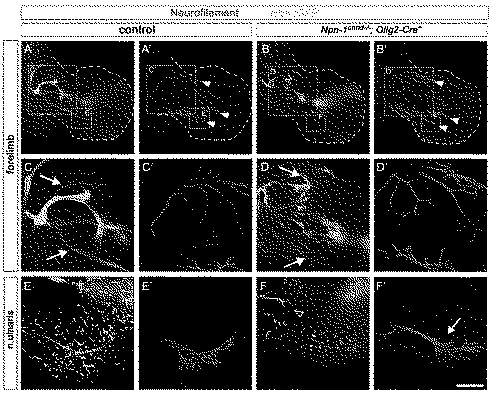
Npn-1 is required in motor neurons for proper fasciculation of LMC projections in the forelimb. Wholemount antibody staining of E12.5 embryos against GFP (green, motor nerves) and neurofilament (red, motor and sensory nerves). Ablation of *Npn-1* from motoneurons leads to severe defasciculation of motor projection to the forelimb in *Npn-1^cond^* ^−*/*−^ *;Olig2-Cre^+^* mutant embryos (B) when compared to wildtype littermate controls (A). A higher magnification of the areas boxed in (A) and (B) reveals that the severe defasciculation of motor nerves in the forelimb is accompanied by absence of several major rami (compare arrows in C and D). Even though the motor projections are severely reduced, the general appearance, positioning, and fasciculation pattern of the sensory trajectory appears normal in *Npn-1^cond^* ^−*/*−^ *;Olig2-Cre^+^* mutant embryos (arrowheads in A', B'). A high magnification in (F') shows normal growth of the sensory compartment of the ulnar nerve (arrow) in absence of motor projections (F) in mutant embryos when compared to controls (E and E'). The open arrowhead in F marks an ectopic motor nerve observed in all mutant embryos. Bar equals 400 µm in (A, B), 100 µm in (C, D), and 80 µm in (E, F). *n* ^mutant^ = 10, *n* ^control^ = 7.

**Figure 3:**
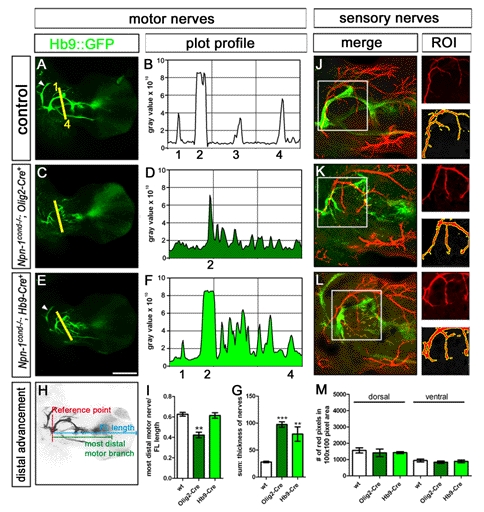
Fasciculation and distal advancement of motor and sensory projections are impaired after ablation of *Npn-1* from motoneurons. Defasciculation of motor nerves was assessed by calculating a plot profile of Hb9::eGFP positive motor projections crossing a virtual line. In control embryos (A and B) four major projections can be seen (1  =  branch of n. radialis, 2 = n. radialis, 3 = n. medianus, 4 = n. ulnaris, arrowhead  =  n. musculocutaneous). In *Npn-1^cond^* ^−*/*−^ *;Olig2-Cre^+^* mutant embryos (C and D) only the remainders of the radial nerve (2) can be assigned to the plot profile, whereas all other motor projections are heavily defasciculated, leading to many peaks along the virtual line. In *Npn-1^cond^* ^−*/*−^ *;Hb9-Cre^+^* mutant embryos defasciculation of motor projections is not as severe as defasciculation caused by *Olig2-Cre*. Therefore, it is possible to assign the small branch of the radial nerve (1), the radial nerve, even though it is defasciculated more distally (2), and the ulnar nerve (4) to the peaks in the plot profile, whereas the median nerve is heavily defasciculated. To quantify the defasciculation of motor fibers, we measured the thickness of the four major motor nerves in wildtype forelimbs (G, 27.8±1.4 SEM) and found significantly increased values in *Npn-1^cond^* ^−*/*−^ *;Olig2-Cre^+^* and *Npn-1^cond^* ^−*/*−^ *;Hb9-Cre^+^* mutants (G, 97.4±5.0 SEM *p*<0.00001 and 79.7±13.4 SEM *p*<0.005, respectively). To quantify the ingrowth of motor axons into the forelimb, the length of the distal-most motor fiber was measured starting from the reference point, and normalized with the length of the forelimb (H). The distal advancement of motor fibers was significantly reduced in *Npn-1^cond^* ^−*/*−^ *;Olig2-Cre^+^* mutant embryos (I, 0.42±0.03, *p*<0.005) compared to control embryos (0.63±0.02), while it was unchanged when *Npn-1^cond^* ^−*/*−^ mice were crossed to the *Hb9-Cre* line (0.61±0.03). Defasciculation of cutaneous sensory nerves was assessed by calculating the number of neurofilament positive red pixels in a given 100×100 pixel region of interest (ROI, white squares in J–M). When compared to control embryos (J), sensory innervation was not altered in *Npn-1^cond^* ^−*/*−^ *;Olig2-Cre^+^* (K) and *Npn-1^cond^* ^−*/*−^ *;Hb9-Cre^+^* (L) mutant embryos in the dorsal or ventral limb. Quantification in (M). *n* = 3 for all genotypes; both limbs were quantified. Scale bar in (E) equals 400 µm for (A, C, E) and 100 µm for (I, J, K).

Absence of Sema3-Neuropilin signaling in all cells, as occurs in the *Npn-1^Sema^* ^−^ mouse line where Sema3A-Npn-1 signaling is abrogated, results in defasciculation of peripheral sensory and motor projections [10]. To determine whether Npn-1 is required cell-autonomously in motoneurons for motor axon fasciculation, we utilized a conditional approach (*Npn-1^cond^*, GeneID:18186, [17]) to selectively remove Npn-1 from this cell type using an *Olig2-Cre* line (GeneID:50913, [18]). In *Npn-1^cond^* ^−*/*−^ *;Olig2-Cre^+^* animals Npn-1 mRNA and protein were strongly reduced in motoneuron cell bodies and axons, respectively, while sensory neurons and axons still expressed unchanged levels of Npn-1 (Figure 1A–E). Motor axons were visualized by also crossing to an *Hb9::eGFP* transgenic mouse line [19]. The formation of peripheral motor and sensory projections was observed in wholemount embryo preparations by GFP fluorescence (motor axons) or expression of neurofilament in absence of GFP (sensory trajectories), respectively. At E12.5, when motor and sensory axons have traversed the plexus region and formed individual nerve branches in the distal limb in the wildtype, motor axons were found to be strongly defasciculated in homozygous *Npn-1^cond^* mice heterozygous for *Olig2-Cre* (Figures 2, S1A,B). These findings were also corroborated when an alternative *Cre* line was used, *Hb9-Cre* (GeneID:15285, [20]), to eliminate Npn-1 from motoneurons (Figure S2). After exiting the spinal cord motor axons converged to form the plexus, however, in *Npn-1^cond^* ^−*/*−^ *;Olig2-Cre^+^* mutants the defasciculation was so severe that hardly any motor fibers reached the distal forelimb (Figure 2B,F, arrowhead). To visualize the degree of defasciculation we measured the pixel intensity along a line perpendicularly crossing the four major motor nerves visible at this developmental time point in the forelimb (Figure 3A). In *Npn-1^cond^* ^−*/*−^ *;Olig2-Cre^+^* animals all nerves are heavily defasciculated and only the radial nerve (number 2 in Figure 3C,D) could still be identified in the plot profile. This also revealed a pronounced defasciculation of motor nerves in the *Npn-1^cond^* ^−*/*−^ *;Hb9-Cre^+^* animals, albeit less severe (Figure 3E,F). To quantify the defasciculation of motor fibers, we measured the thickness of the four major motor nerves in the forelimbs and found significantly increased values in *Npn-1^cond^* ^−*/*−^ *;Olig2-Cre^+^* and *Npn-1^cond^* ^−*/*−^ *;Hb9-Cre^+^* mutants when compared to wildtype littermates (Figure 3G). Since the distal advancement of motor axons in the forelimb appeared to be reduced in *Npn-1^cond^* ^−*/*−^ *;Olig2-Cre^+^* mutants, we quantified the ingrowth of motor axons into the forelimb by measuring the length of the distal-most motor fiber relative to the length of the forelimb (Figure 3H). We found that the advancement of motor axons was significantly reduced in *Npn-1^cond^;Olig2-Cre^+^* embryos (0.42±0.03 SEM) compared to wildtype littermates (0.63±0.02, *p*<0.005) while the distal advancement was normal in *Npn-1^cond^* ^−*/*−^ *;Hb9-Cre^+^* animals (Figure 3I). Surprisingly, the formation of sensory trajectories was unaffected by defasciculation or stunted growth of motor nerves in *Npn-1^cond^* ^−*/*−^ *;Olig2-Cre^+^* mutants (Figure 2B',D'). This was particularly obvious with the sensory part of the ulnar nerve that was formed normally even though the motor nerve did not extend as far as in control animals (Figure 2E',F', arrow). We quantified the defasciculation of sensory projections by counting the number of neurofilament positive pixels in a defined region of interest and found no difference in *Npn-1^cond^* ^−*/*−^ *;Olig2-Cre^+^* or *Npn-1^cond^* ^−*/*−^ *;Hb9-Cre^+^* mutants when compared to wildtype littermates (Figure 3J–M). Npn-1 is also expressed in a large majority of LMC neurons at lumbar level and indeed we observed a very similar phenotype in the hindlimb where, after removal of Npn-1 from motoneurons, motor projections were significantly defasciculated while the sensory branching pattern was established normally (Figure S3, quantification in Figure S4). Interestingly, not only LMC projections to the limbs were affected by selective removal of Npn-1 in motoneurons, but also other motor projections, as exemplified by the innervation of intercostal musculature originating from neurons of the lateral aspect of the medial motor column (MMCl, Figure S5). In contrast to wildtype intercostal nerves, which are tightly bundled (Figure S5A,B), these nerves are strongly defasciculated in *Npn-1^cond^* ^−*/*−^ *;Olig2-Cre^+^* mutant mice with many axon fascicles crossing between major nerve branches (Figure S5D, arrowheads). The mean number of such crossings in mutant embryos was almost 30-fold higher than in control littermates (14.17±4.1 versus 0.5±0.5 crossings per embryo, respectively, *p*<0.005, Figure S5E). Interestingly, defasciculated intercostal axons were also observed later in embryonic development at E15.5 (Figure S6). These data indicate that Npn-1 is required cell-type autonomously in motoneurons for proper fasciculation of motor trajectories. They further suggest that peripheral sensory projections are established correctly, even if the motor projections are severely defasciculated in the distal fore- and hindlimb due to loss of Npn-1 function.

### Npn-1 Is Required in Motoneurons Cell-Autonomously for Accurate Dorsal-Ventral Guidance of LMC Axons

**Figure 4:**
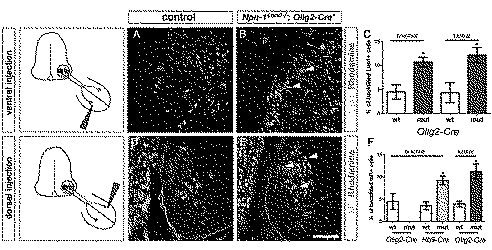
LMC projections are misrouted when Npn-1 is removed from motor neurons. Retrograde tracing of ventrally and dorsally projecting LMC neurons by injection of dextran-coupled Rhodamine into limb musculature of E12.5 embryos. Injection of fluorescent tracer into the ventral musculature of *Npn-1^cond^* ^−*/*−^ *;Olig2-Cre^+^* mutant embryos shows a significant increase of aberrantly projecting Lim1 positive neurons of the LMCl at lumbar (*n* = 4, 12.16%±1.59%, *p* ^lumbar^ = 0.03) and brachial levels (*n* = 4, 10.71±0.99, *p* ^brachial^  =  0.01; arrowheads in B, C) when compared to controls (*n* = 3, 4.33%±2.11% and *n* = 4, 4.48%±1.5%, respectively). In *Npn-1^cond^* ^−*/*−^ *;Olig2-Cre^+^* mutant embryos 11.25%±1.66% (*p* = 0.01) of dorsally backfilled neurons were Isl1 positive and thus projecting aberrantly at lumbar levels (*n* = 3, arrowheads in E) compared to only 3.9%±0.65% in littermate controls (*n* = 3, D, F). At brachial levels, dorsal backfills did not lead to labeled motor neurons. Analysis of *Npn-1^cond^* ^−*/*−^ *;Hb9-Cre^+^* mutant embryos shows a significant number (9.21%±0.97%, *p* = 0.012, *n* = 3) of aberrantly projecting LMCm motor neurons to dorsal musculature (F) at brachial levels when compared to littermate control embryos (*n* = 3, 3.5%±0.88%). Bar equals 45 µm in (A, B, D, E).

Defasciculation of motor projections as caused by removal of Npn-1 from motoneurons could affect the stereotypical dorsal-ventral choices made by LMC axons. We therefore examined whether cell-type-specific removal of Npn-1 affected this guidance decision. We retrogradely labeled motoneuron cell bodies by injecting rhodamin-coupled dextran into either ventral or dorsal forelimb muscles of E12.5 embryos and then assessed the presence of retrogradely transported dextran in the cell bodies of Lim1-positive LMCl motoneurons projecting to dorsal musculature or Isl1-positive LMCm motoneurons projecting to ventral limb muscles, respectively. In wildtype embryos, only very few motoneurons that were retrogradely labeled from ventral fore- or hindlimb muscle injections expressed Lim1 (Figure 4A,C). In contrast, significantly more ventrally labeled rhodamin-positive motoneurons expressed Lim1 in *Npn-1^cond^* ^−*/*−^ *;Olig2-Cre^+^* mutant embryos (Figure 4B,C, arrowheads). Thus, removal of Npn-1 from motoneurons leads to misrouting of LMCl axons to the ventral half in both fore- and hindlimb. Analysis of the fidelity of LMCm projections by retrograde labeling from dorsal limb muscles revealed that 11.25%±1.66% of lumbar LMCm axons aberrantly projected to the dorsal limb when Npn-1 was removed from motoneurons (Figure 4E,F). Due to the shortened ingrowth of motor axons in *Npn-1^cond^;Olig2-Cre^+^* mutants none of the dorsal backfills at brachial levels resulted in any retrogradely traced neurons (Figure 3I). We therefore assessed the fidelity of the brachial dorsal-ventral choice also in *Npn-1^cond^* ^−*/*−^ *;Hb9-Cre^+^* mutants where motor axons, though defasciculated, are found at comparable distal positions in the forelimb as in control littermates (Figure 3I). Retrograde tracing from dorsal forelimb musculature revealed a significant number of misguided Isl1 positive LMCm neurons in these mutant embryos (Figure 4F). Together, these data show that decreased fasciculation caused by motoneuron-specific loss of Npn-1 leads to pathfinding errors at the dorsal-ventral choice point.

### Ablation of Npn-1 from Sensory Neurons Leads to Defasciculation of Sensory and Motor Projections

**Figure 5:**
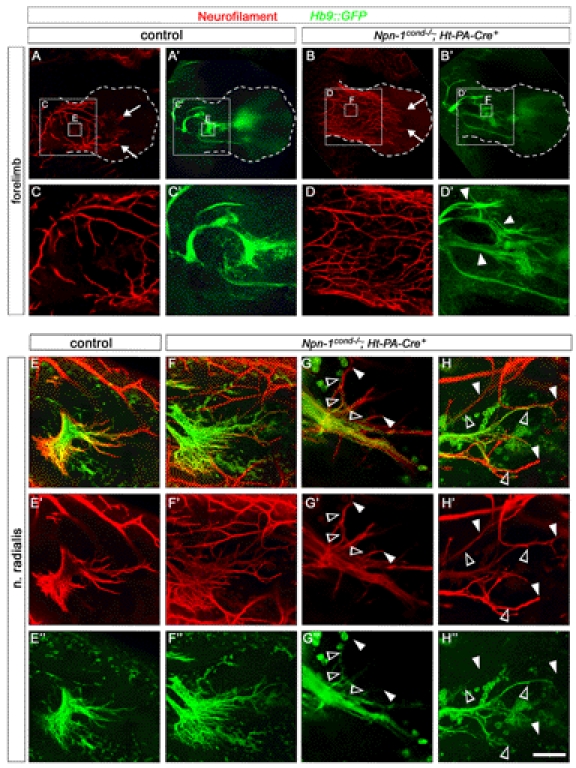
Removal of Npn-1 from sensory neurons leads to defasciculation of sensory and motor projections to the limbs. Wholemount antibody staining of E12.5 embryos against GFP (green, motor nerves) and neurofilament (red, motor and sensory nerves). Ablation of *Npn-1* from sensory neurons leads to severe defasciculation and exuberant growth of sensory projections to the forelimb in *Npn-1^cond^* ^−*/*−^ *;Ht-PA-Cre^+^* mutant embryos (arrows in B, D) when compared to littermate controls (arrows in A, C). A higher magnification of the boxed areas in (A) and (B) reveals that the severe defasciculation of sensory projections is associated with defasciculation of major motor nerve trunks in the forelimb (arrowheads in D'). A high magnification of the radial nerve shows aberrant projections of motor axons (open arrowheads in G, H) that are always preceded by an aberrantly projecting sensory axon (arrowheads in G, H). *n* ^mutant^  =  7, *n* ^control^  =  8.

**Figure 6:**
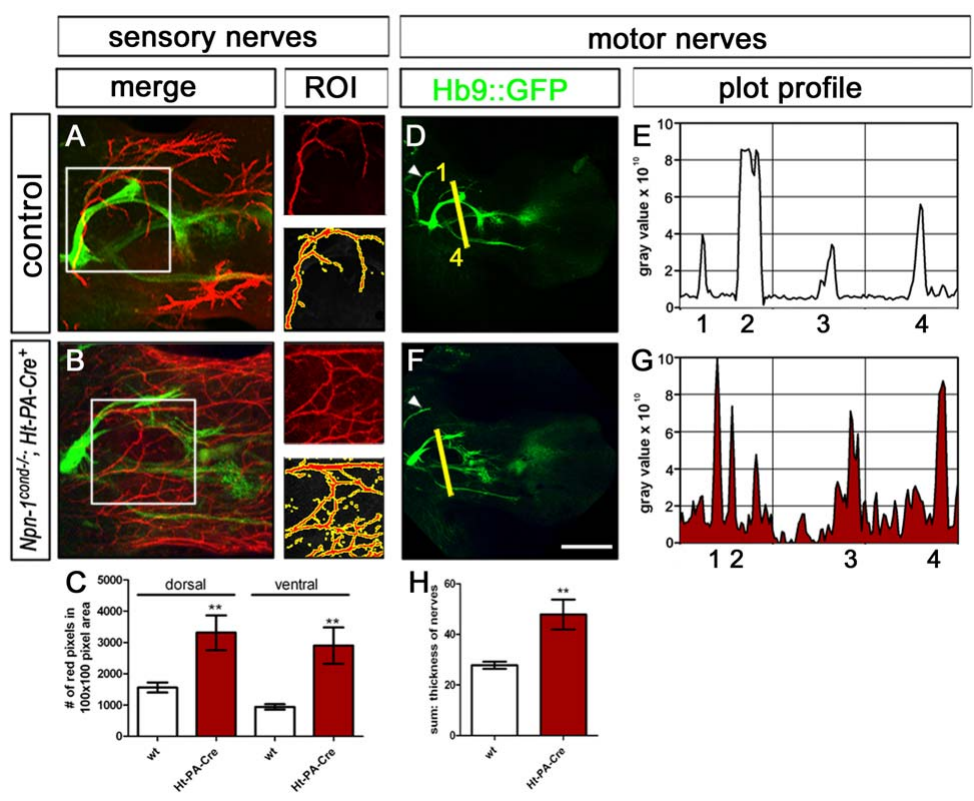
Fasciculation of sensory and motor projections is affected after ablation of *Npn-1* from sensory neurons. Defasciculation of cutaneous sensory nerves was assessed by calculating the number of neurofilament positive red pixels in a given 100×100 pixel region of interest (ROI). When compared to control embryos (A) this number is significantly increased in *Npn-1^cond^* ^−*/*−^ *;Ht-PA-Cre^+^* mutant embryos (B, C) for the cutaneous innervation of the dorsal and ventral limb (*p* ^dorsal^<0.005, *p* ^ventral^<0.005). Defasciculation of motor nerves was assessed by calculating a plot profile of Hb9::eGFP positive motor projections crossing a virtual line (1  =  branch of n. radialis, 2 = n. radialis, 3 = n. medianus, 4 = n. ulnaris, arrowhead  =  n. musculocutaneous). When compared to control embryos (D, E) all four nerve branches can be found in *Npn-1^cond^* ^−*/*−^ *;Ht-PA-Cre^+^* mutant embryos, however, at slightly inappropriate positions to each other, and defasciculated fibers can be found in between major nerve branches. Quantification of the defasciculation of motor projections by measuring the thickness of the four major nerves revealed a significant increase in *Npn-1^cond^* ^−*/*−^ *;Ht-PA-Cre^+^* mutant embryos (H, 47.9±5.98 SEM) when compared to wt embryos (27.8±1.4 SEM, *p*<0.0001. The scale bar in (F) equals 100 µm for (A, B) and 400 µm for (D, F). *n* = 3 for all both genotypes; both limbs were quantified.

**Figure 7:**
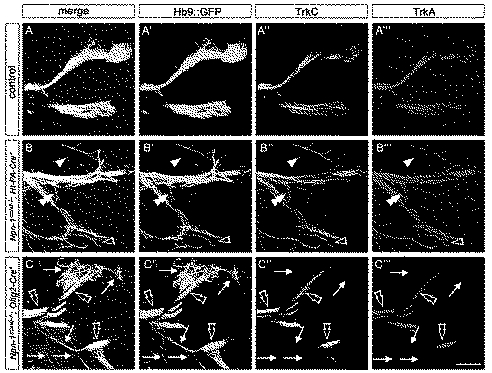
Defasciculation of motor fibers is accompanied by defasciculation of TrkA and TrkC positive fibers in *Npn-1^cond^* ^−*/*−^ ***;Ht-PA-Cre^+^***, but not in *Npn-1^cond^* ^−*/*−^ *;Olig2-Cre^+^* **mutant embryos.** Fluorescent immunohistochemistry shows that nociceptive TrkA positive (red) and proprioceptive TrkC positive (white) fibers accompany motor nerves (*Hb9::GFP*, green) on their way into the distal limb (control in A). In *Npn-1^cond^* ^−*/*−^ *;Ht-PA-Cre^+^* mutant embryos defasciculation of motor projections is only observed in combination with severe defasciculation of sensory trajectories, either axons positive for TrkA or TrkC (empty arrowhead and arrowhead, respectively, in B), or fibers positive for both TrkA and TrkC (double arrowhead in B). In *Npn-1^cond^* ^−*/*−^ *;Olig2-Cre^+^* mutant embryos no defasciculated sensory axons were observed even in areas with clear defasciculation of motor projections (arrows in C). Sensory axons grow rather fasciculated and do not seem to be affected by defasciculation of motor pathways (empty double arrowheads in C). Scale bar equals 100 µm for all panels.

We next assessed the consequences of deletion of *Npn-1* in sensory neurons by crossing *Npn-1^cond^* mice with a transgenic line expressing Cre recombinase under the control of the *human tissue plasminogen activator* promoter (*Ht-PA-Cre*). This line targets all known derivatives of neural crest cells and, hence, also sensory neurons of the DRG but not central nervous system (CNS) neurons [21]. In *Npn-1^cond^* ^−*/*−^ *;Ht-PA-Cre^+^* animals we found reduced levels of Npn-1 mRNA and protein in sensory neurons and axons of the DRG, respectively, while motor neurons and axons expressed unchanged amounts of Npn-1 (Figure 1F–J). At E12.5, we found a pronounced defasciculation of sensory projections innervating both the fore- and hindlimbs and aberrant, exuberant growth when compared to wildtype embryos (Figure 5A,C and B,D, arrows; Figure S3G',H'). We quantified the degree of defasciculation of sensory projections to the dorsal or ventral forelimb and found a dramatic increase in *Npn-1^cond^* ^−*/*−^ *;Ht-PA-Cre^+^* mutants compared to wildtype littermates (Figure 6A–C). Surprisingly, this incorrect pattern of sensory projections was accompanied by defasciculation of motor axons: several major motor branches, particularly the radial and median nerves to the forelimb, were defasciculated (Figure 5B',D', arrowheads). To visualize this phenotype we generated a plot profile by measuring the pixel intensity of the Hb9::eGFP staining along a line perpendicularly crossing the four major nerves in the E12.5 forelimb (Figure 6D–G). We found that removal of Npn-1 from sensory neurons leads to a defasciculation of motor trajectories in *Npn-1^cond^* ^−*/*−^ *;Ht-PA-Cre^+^* mutants, particularly of the n. radialis. We quantified the degree of defasciculation by measuring the thickness of the four major motor nerves and found a significantly increased value in *Npn-1^cond^* ^−*/*−^ *;Ht-PA-Cre^+^* mutants when compared to wildtype littermates (Figure 6H), which was, however, less severe than the defasciculation caused by ablation of Npn-1 from motor neurons (Figure 3C–G). Interestingly, these motor branches, while defasciculated, still developed in roughly appropriate relative positions to each other and the distal advancement of motor axons was normal (Figure S4). At higher magnification, we noted that defasciculated motor projections were always accompanied by sensory axons that preceded motor axons (e.g. in the radial nerve, Figure 5G,H, arrowheads). At the same time, many defasciculated sensory axons were observed (Figure 5F–H). When we distinguished proprioceptive from nociceptive sensory projections by TrkC or TrkA immunohistochemistry, respectively, we found that in *Npn-1^cond^* ^−*/*−^ *;Ht-PA-Cre^+^* mutants, defasciculated motor axons follow either TrkC- or TrkA-positive fibers (Figure 7B, arrowhead and open arrowhead, respectively). At this stage, Npn-1 is expressed in the majority of TrkA-positive DRG neurons, but also in a significant number of TrkC-positive sensory neurons and is therefore in a position to mediate fasciculation of nociceptive and proprioceptive axons (Figure S7). The phenotype observed in *Npn-1^cond^* ^−*/*−^ *;Ht-PA-Cre^+^* is in stark contrast to the situation in *Npn-1^cond^* ^−*/*−^ *;Olig2-Cre^+^* mutants where Npn-1 is removed from motoneurons. Here, defasciculated motor axons are not followed by nociceptive or proprioceptive fibers (Figure 7C, arrows).

In the hindlimb, after removal of Npn-1 from sensory neurons, both the peroneal and tibial nerves were defasciculated (Figure S3G,H). In addition, we found guidance defects of motor axon bundles aberrantly turning back towards the proximal limb (Figure S3H, open arrowhead, observed in two out of five embryos in addition to defasciculated motor and sensory projections). Also, the innervation of intercostal muscles was affected by the ablation of *Npn-1* from sensory neurons in *Npn-1^cond^* ^−*/*−^ *;Ht-PA-Cre^+^* mutants. Intercostal nerve branches were severely defasciculated and axon fascicles frequently crossed between the main branches (Figure S5F,G, arrowheads), a phenotype that was only rarely observed in control littermates (13.25±1.25 versus 0.4±0.24, respectively, *p*<0.0001). From this we conclude that Npn-1 is required in sensory neurons for proper fasciculation of sensory projections. In addition, defasciculation of sensory axons by removal of Npn-1 leads to a compromised development of motor trajectories.

To address whether the less severe defasciculation of motor projections caused by absence of Npn-1 from sensory neurons also affects the dorsal-ventral guidance decision of motor axons at the base of the limb, we retrogradely traced motor projections to the dorsal and ventral mesenchyme in fore- and hindlimb of *Ht-PA-Cre* mutants. Our data show that medial and lateral LMC neurons at brachial and lumbar levels project correctly to the ventral or dorsal limb mesenchyme, respectively (Figure S8). We thus conclude that the defasciculation of motor axons induced by removal of Npn-1 from sensory neurons does not lead to guidance errors at the dorsal-ventral choice point at the base of the limb.

### Axon Bundling Before the Plexus Determines Distal Axon Fasciculation

**Figure 8:**
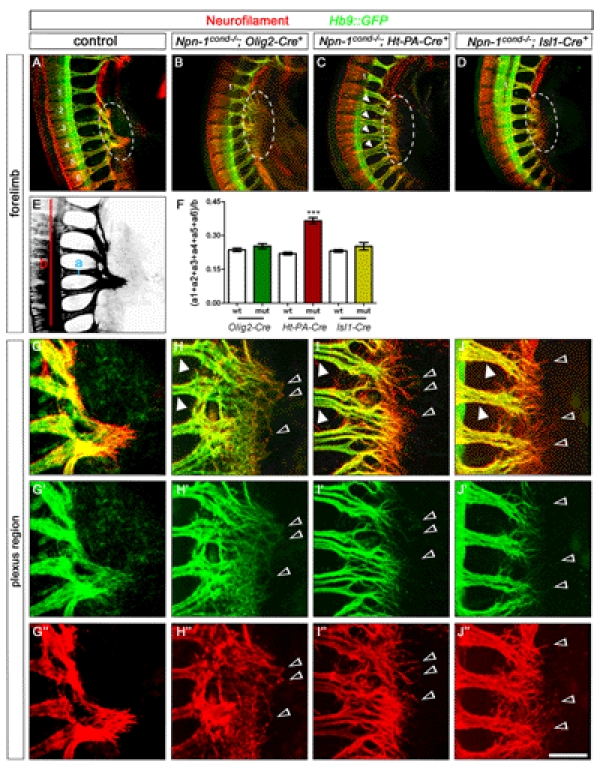
Fasciculation before the plexus determines the fasciculation in the distal limb. Side view of forelimb plexi of E10.5 wholemount embryos stained against GFP (green, motor nerves) and neurofilament (red, motor and sensory nerves), numbers 1–6 in (A) mark the spinal nerves contributing to the forelimb plexus, and the plexus region is encircled with a white dashed line. Elimination of *Npn-1* selectively from motoneurons (*Olig2-Cre*) leads to defasciculation of motor nerves in the plexus region (B and H), however fasciculation before the plexus region was not significantly altered (*n* = 4, B, arrowheads in H). Sensory nerves stay behind motor axons in the plexus (open arrowheads in H). Note that all defasciculated and disorganized axons in the plexus region are positive for Hb9::GFP, indicating that these are motor axons. In *Npn-1^cond^* ^−*/*−^ *;Ht-PA-Cre^+^* mutant embryos (C) motor and sensory axons do not converge to form a plexus as in control embryos (A), but sensory projections are further advanced than motor axons (open arrowheads in I). Motor and sensory trajectories are defasciculated already before the plexus region (*n* = 6, arrowheads in C and I). When *Npn-1* is ablated from both motor and sensory neurons (*Npn-1^cond^* ^−*/*−^ *;Isl1-Cre^+^*) mutant embryos show an intermediary phenotype with sensory and motor projections defasciculated in the plexus region, however only two-thirds of the mutant embryos exhibit pre-plexus defasciculation (*n* = 6, D). For quantification of pre-plexus defasciculation (F) the thickness of the six individual spinal nerves (a1–a6) contributing to the forelimb plexus was measured, added up, and divided by the length of the area occupied by these spinal nerves (b) for both forelimb regions of three embryos (E). The fasciculation coefficient was significantly increased in *Npn-1^cond^* ^−*/*−^ *;Ht-PA-Cre^+^* mutant embryos when compared to littermate controls (*p* ^Olig2-Cre^  =  0.2690, *p* ^Ht-PA-Cre^<0.0001, *p* ^Isl-Cre^  =  0.2182). Bar graph equals 500 µm in (A–D) and 150 µm in (G–J).

After motor and sensory axons exit the spinal cord and DRG, respectively, they converge in the plexus region where sorting into new target-specific bundles occurs. We monitored the formation of distinct projections to the forelimb at E10.5 when wildtype motor and sensory projections have reached the plexus but have not yet navigated through this dorsal-ventral choice point (Figure 8). Motoneuron-specific deletion of *Npn-1* resulted in pronounced defasciculation of motor axons in the plexus region (circled area in Figure 8B; all defasciculated axons were stained for GFP indicating their motor origin). Interestingly, defasciculation in *Npn-1^cond^* ^−*/*−^ *;Olig2-Cre^+^* mutants is limited to the plexus region: after exiting the spinal cord, nerve bundles projected directly towards the plexus in a fasciculated manner (Figure 8H, arrowheads indicate fasciculated spinal nerves before the plexus). In contrast, elimination of *Npn-1* from sensory neurons resulted in pronounced defasciculation of both motor and sensory projections before these axons reach the plexus as well as in the plexus region in *Npn-1^cond^* ^−*/*−^ *;Ht-PA-Cre^+^* mutants (sensory axons were identified by staining positive for neurofilament and absence of GFP immunoreactivity, Figure 8C,I). Interestingly motor and sensory axons are spread out over a wider area than in control embryos before they reach the plexus (Figure 8I, arrowheads). Within the plexus region, however, the tips of sensory axons are further advanced than motor fibers and are severely defasciculated (Figure 8I,I’,I’’, open arrowheads). To quantitatively compare the degree of pre-plexus defasciculation between different genotypes, we calculated a fasciculation coefficient by measuring the width of the spinal nerves contributing to the forelimb at their narrowest point and relating it to the total rostro-caudal length of the analyzed segments (“a” and “b”, respectively, in Figure 8E). The fasciculation coefficient was significantly higher only when *Npn-1^cond^* mice were crossed to *Ht-PA-Cre*, while there was no difference in *Npn-1^cond^* ^−*/*−^;*Olig2-Cre^+^* mutants (Figure 8F, *p*<0.01 and *p*<0.2, respectively, see figure legend). Deletion of *Npn-1* from both sensory and motoneurons by combining the *Npn-1^cond^* allele with an *Isl1-Cre* transgenic line [22] resulted in an intermediary phenotype (Figure 8D,J): while spinal nerves approach the plexus in a normally fasciculated way, a strong defasciculation was observed within the plexus with sensory axons leading motor fibers (Figure 8J,J' open arrowheads).

**Figure 9:**
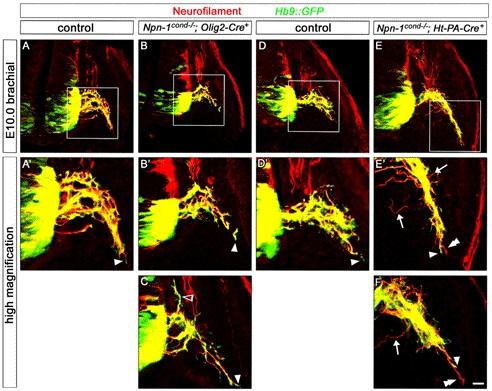
Sensory axons overtake motor axons after removal of Npn-1 in sensory neurons. Coronal sections of E10.0 (Theiler stage 16, 30–32 somites) embryos, staining against Hb9::eGFP and neurofilament. In control embryos, GFP expression was found all along motor axons into the distal-most tips of outgrowing trajectories (arrowhead in A' and C), indicating that motor axons lead the spinal nerve projection. In *Npn-1^cond^* ^−*/*−^ *;Olig2-Cre^+^* mutant embryos some motor axons choose a wrong trajectory, turning dorsally into the DRG (open arrowhead in C), however GFP-positive motor axons are observed in the tips of the leading axons growing towards the limb (arrowheads in B' and C). In *Npn-1^cond^* ^−*/*−^ *;Ht-PA-Cre^+^* mutant embryos sensory axons defasciculate from the forming spinal nerve (arrows in E' and F, note the missing GFP expression in those nerves, therefore classified as sensory axons). Note that sensory axons outgrow motor axons (double arrowheads in E' and F), indicating that sensory nerves overtake motor axons already on the way towards the plexus. Bar equals 20 µm for (A–E) and 10 µm for (A'–E', C, and F).

These data suggest that the state of sensory axon fasciculation before entering the plexus region influences motor axon fasciculation before, within, and after the plexus. We therefore analyzed the sensory-motor projections at E10.0, when motor axons have left the spinal cord and joined the sensory fibers but have not yet reached the plexus region. We found that the sensory projection is defasciculated already at this early timepoint in *Npn-1^cond^* ^−*/*−^;*Ht-PA-Cre^+^* mutants and that sensory axons are frequently further advanced than motor fibers, a behavior that was never observed in wildtype littermate controls (Figure 9D–F). In contrast, motor and sensory projections are indistinguishable in *Npn-1^cond^* ^−*/*−^;*Olig2-Cre^+^* mutants compared to control embryos (Figure 9A–C). In particular, motor axons exit the spinal cord in a normally fasciculated manner in mutant embryos. In a small number of embryos, at brachial levels, motor axons were found to turn dorsally after exiting the spinal cord and to project into the DRG (Figure 9C, open arrowhead). Together, our data suggest that removal of Npn-1 from sensory neurons breaks the tight coupling of sensory and motor axons and allows for sensory fibers to overtake motor axons.

### Elimination of Sensory or Motor Neurons Impairs the Correct Formation of Both Projections Distinctly

**Figure 10:**
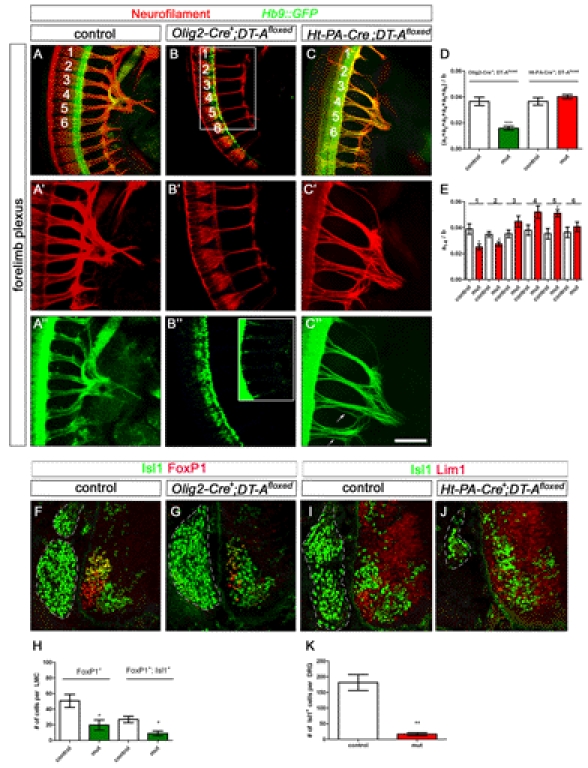
Elimination of motor or sensory neurons influences fasciculation and timing of peripheral projections. Wholemount antibody staining of E10.5 embryos (A–C'') against GFP (green, motor nerves) and neurofilament (red, motor and sensory nerves). Ablation of either developing motor (*Olig2-Cre^+^*; B) or sensory neurons (*Ht-PA-Cre^+^*; C) upon DT-A expression results in a delay in axonal ingrowth in the forelimb plexus. Interestingly, sensory axons are capable of navigating to the plexus region if motor axons are completely absent or severely reduced (B' and B'' and inlay, respectively). Reduction of sensory projections in *Ht-PA-Cre^+^;DT-A ^floxed^* embryos causes thinned or absent sensory projections (spinal nerves 1, 2 in C) as well as defasciculation of sensory and motor axons of more posterior nerves (arrows in C''). For quantification of pre-plexus defasciculation and axonal thinning, the diameter of the six spinal nerves of the plexus was measured for both forelimbs of four control and six mutant embryos. Either the sum of the six spinal nerves or of individual spinal nerves (a) was normalized to the length of the occupied area (b). The fasciculation coefficient was significantly decreased in *Olig2-Cre^+^;DT-A ^floxed^* mutant embryos but not in *Ht-PA-Cre^+^;DT-A ^floxed^* when compared to controls (D; p^Olig2-Cre^<0.001, p^Ht-PA-Cre^  =  0.2602). Analysis of the fasciculation status of the individual spinal nerves in *Ht-PA-Cre^+^;DT-A ^floxed^* embryos revealed either thinning (nerves 1 and 2) or defasciculation (nerve 5) depending on their anterior-posterior position in the forelimb (E). Immunohistochemical quantification of the partial elimination of motor (*Olig2-Cre^+^;*F–H) or sensory neurons (*Ht-PA-Cre^+^* I–K) upon tissue-specific expression of lethal *diphteria toxin A fragment (DT-A ^floxed^)* shows that the number of LMCm (FoxP1^+^;Isl1^+^; *p*<0.05) and LMCl neurons (FoxP1^+^; *p*<0.05) is significantly decreased in *Olig2-Cre^+^;DT-A^floxed^* mutants (G) when compared to littermate controls (F) at E11.5 (H, *n* ^control^  =  3; *n* ^mutant^  =  3). The number of sensory neurons in the DRG is significantly decreased in *Ht-PA-Cre^+^;DT-A^floxed^* mutants (J,) when compared to controls (I) per DRG (K, Isl1^+^ cells per DRG; *n* ^control^  =  3; *n* ^mutant^  =  3; *p*<0,005). Bar equals 400 µm in (A–C'') and 50 µm in (F–J).

**Figure 11:**
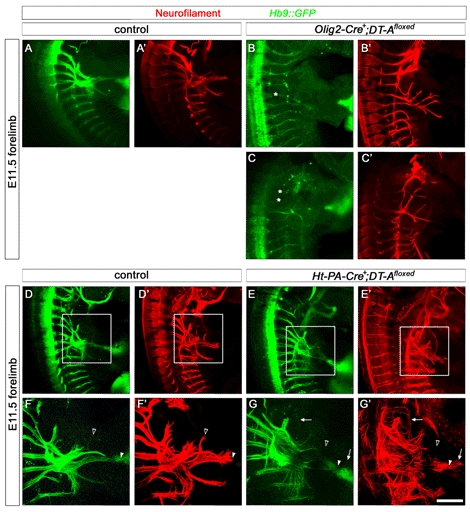
Elimination of motor or sensory neurons impairs the formation of the untouched axonal projection. Wholemount antibody staining of E11.5 embryos against GFP (green, motor nerves) and neurofilament (red, motor and sensory nerves). Partial elimination of motor (*Olig2-Cre^+^)* or sensory (*Ht-PA-Cre^+^*) neurons was achieved by tissue-specific expression of lethal *DT-A (DT-A^floxed^)*. Ablation of motoneurons (*Olig2-Cre^+^;DT-A^floxed^*, B, C) impairs the formation of sensory trajectories and their branching behavior, whereby both increased and decreased branching was observed (B' and C'). The reduced number of sensory neurons in *Ht-PA-Cre^+^;DT-A^floxed^* mutants (E, E') leads to absence of some anterior spinal nerve and branches in the limb (open arrowhead in G') as well as a dramatic defasciculation of remaining sensory axons (arrows, G') when compared to wildtype littermates (D, F). Motor axons are also defasciculated and less far advanced than sensory axons (arrowhead in G, G'). Bar equals 400 µm in (A–E) and 200 µm in (F, G).

To investigate whether sensory fibers require motor axons at all for the correct formation of their peripheral projection we utilized a genetic approach to deplete motoneurons. Crossing a conditional *diphteria toxin fragment A* (*DT-A*) transgenic line [23] with *Olig2-Cre* mice resulted in partial removal of motoneurons (Figure 10F–H). Motoneurons hardly sent out any axons at all (see inset in Figure 10B''). Interestingly, sensory axons were able to project towards the plexus region, however at this developmental stage, their growth appeared delayed as the individual spinal nerves did not join together to form a distinct plexus and no axons were observed entering the forelimb (Figure 10B'). At E11.5 very few motoneurons sent out axons at all, and some segmental spinal nerve branches were completely absent (Figure 11A–C, asterisks). Interestingly, sensory projections developed, although reduced in thickness, with a considerable variation in the fidelity of the normally stereotypical projection patterns. In different animals, both increased and decreased branching frequencies in the distal limb were observed (Figure 11B',C'). In addition, the number of DRGs contributing to the innervation was reduced in the mutant embryos where only sensory branches from 3 to 4 DRGs were observed to project towards the forelimb.

We next analyzed the behavior of motor axons when sensory neurons were eliminated by combining the *Ht-PA-Cre* line with the floxed *DT-A* mice. At E10.5, the number of sensory neurons in the DRG was dramatically reduced in *Ht-PA-Cre^+^:DT-A^floxed^* mutants (Figure 10I–K). Reminiscent of our earlier experiments where removal of Npn-1 from sensory neurons caused defasciculation of motor axons (Figures 5 and 8), we observed defasciculated motor projections prior to the plexus region (Figure 10C'', arrows). At E11.5 the remaining sensory axons were defasciculated and single peripheral branches missing (Figure 11G', open arrowhead). In addition, certain spinal nerves, particularly the most anterior nerve, did not join the plexus and contribute to the peripheral limb projection. Interestingly, motor axons were also dramatically defasciculated and not as far advanced as the remaining sensory axon (Figure 11G, arrowhead).

Together, these data support the notion that motor axons are required at an early stage in sensory trajectory development, when sensory axons need to find their way to the plexus. At later stages, after having navigated the plexus, sensory fibers become independent of motor axons for their projection to the distal limb, hence defasciculation or even complete absence of motor axons has a lesser influence on the patterning of sensory trajectories. Our data also indicate that motor projections are defasciculated and impaired in the distal advancement by absent or reduced sensory trajectories.

## Discussion

Investigations into how growing axons navigate the environment to reach their targets during development have yielded a growing list of molecular cues and corresponding receptors that determine guidance decisions [24]. A strong focus of these studies has been to understand growth cone behavior at defined positions, so called choice points, where growth cones of nerve fibers pause, explore their environment, and, subsequently, in response to local cues, resume their growth in the appropriate direction. The concept of pioneering axons that lay down the landscape for later following fibers has been described for many vertebrate systems from retinotectal to callosal projections [25]–[28]. During development, however, only a small number of pioneer axons grow into “uncharted” territories, the vast majority of fibers orient their growth patterns along axon tracts already laid out by preceding fibers. Nevertheless, in order to establish correct trajectories these fibers cannot simply follow existing trajectories but need to make appropriate decisions about which branch to follow, and when to form new rami. These interactions with existing fiber bundles clearly require strictly regulated events of selective fasciculation and de-fasciculation, the molecular basis of which are in most cases not well understood. In this study we have explored the coordinated growth and fasciculation of sensory and motor fibers during the establishment of limb innervation and in particular the role of a well-characterized guidance receptor, Npn-1, in the interaction of these axonal populations.

### Fasciculation of Motor Axons and Its Role in Establishing Peripheral Sensory Trajectories

In the spinal nerve, sensory axons from the DRG and motor axons from the ventral horn of the spinal cord converge to establish conjoined trajectories to their respective peripheral targets. Whether and to what degree sensory axons depend on motor projections in the formation of their peripheral projection patterns has been controversial. Early surgical removal of motoneurons in the embryonic chick resulted in abnormal patterning of sensory trajectories [15], while eliminating motoneurons after neural crest cells have coalesced into DRG had no obvious impact on the formation of sensory projections to muscles [16]. We used a genetic approach to partially eliminate motoneurons. Our findings indicate that sensory axons depend on a minimal scaffolding of motor fibers to correctly establish their trajectory, however very few fibers suffice to allow for sensory projections to form. The guidance receptor Npn-1 is expressed in LMC neurons at brachial and lumbar levels as well as in sensory neurons of the DRG and therefore presents itself as a plausible candidate to mediate these inter-axonal interactions. When we removed Npn-1 specifically from motoneurons, we found a dramatic defasciculation of motor axons in the plexus region and in the distal limb accompanied by a reduced extension into the distal limbs (Figures 2, 3). The formation of the sensory trajectories, however, was unaffected with respect to fasciculation and localization of individual nerve branches. One possible explanation for this normal development is that the sensory neurons correctly present Npn-1 on the cell surface and Sema3A expressed in adjacent tissues efficiently promotes sensory axon fasciculation through a surround repulsion mechanism [1],[10],[29]. In addition, even though defasciculated, motor axons are still present in the proximal limb and might nonetheless facilitate sensory fiber growth through the plexus region by providing an aligned substrate [15]. Interestingly, while removal of Npn-1 from motoneurons leads to dramatic defasciculation of motor axons in the plexus and distal limb, motor fibers remain normally fasciculated in the segment of their trajectory preceding the plexus. Recently, it was shown that Drosophila axons in culture are intrinsically divided into two compartments and that the guidance receptors Derailed and ROBO2/3 are differentially localized to proximal and distal compartments, respectively [30]. Diffusion barriers preventing the exchange of specifically localized membrane proteins have also been described in cultured mammalian neurons, prohibiting the exchange of membrane proteins between the somatodendritic and axonal compartments at the axon initial segment, and between the proximal and distal segments of the axon [31]. The concept of diffusion barriers regulating the presence and/or concentration of cell surface molecules within defined segments of axonal trajectories is an attractive model to explain differential sensitivities to guidance cues of growing axons for various stages of development. It will be interesting to determine whether such diffusion barriers exist at specific choice points of the spinal motor projection, such as the base of the limb, and regulate Npn-1 localization preferentially to the distal motor axon shaft, hence controlling motor axon fasciculation within and beyond the plexus region.

The defasciculation caused by removal of Npn-1 from motoneurons is also accompanied by defects in the selection of the dorsal-ventral trajectory after the plexus. Interestingly, this phenotype is less severe compared to dorsal-ventral pathfinding errors that were observed after complete interruption of Sema3-Npn-1 signaling by transgenic replacement of a mutated Npn-1 receptor that is incapable of Sema3 binding [5]. A residual function might be maintained by non-quantitative Cre-mediated recombination at the *Npn-1* locus and a subset of motoneurons still expressing Npn-1. The wide scattering of motor axons in the plexus area likely interferes with the required interaction and disrupts the assembly of correct bundles of axons traveling to the same peripheral targets. Hence, pre-target axon sorting is hampered and the establishment of the topographic projections of lateral LMC axons to the dorsal limb and medial LMC axons to the ventral limb is impaired [5].

Most of our analysis was done at early embryonic stages and thus raises the question of whether the defects in fasciculation that were induced by the removal of Npn-1 are transient or maintained into later developmental or even adult stages. While a previous study reported the correction of aberrant projections in embryonic *Sema3A* mutants [32], we found persistent defasciculation of intercostal nerves after removal of Npn-1 (Figure S6) and in mice with non-functional binding of Sema3 to Npn-1 [33] at least through E15.5. Unfortunately, due to poor reagent penetration and increasingly higher GFP background staining of the Hb9::eGFP line, an analysis of the deeper motor axons of the forelimb is not feasible at these late embryonic stages. However, anatomical, electrophysiological data and behavioral analysis of locomotor skills of adult *Npn-1^cond^* ^−*/*−^ *;Olig2-Cre^+^* mutants demonstrate that defects in motor connectivity persist ([33], Soellner and Huber, unpublished), suggesting that indeed mutant phenotypes are maintained at least to some degree.

### Sensory Projections Mediate Fasciculation, But Not Dorsal-Ventral Choice of Motor Axons through Npn-1

**Figure 12:**
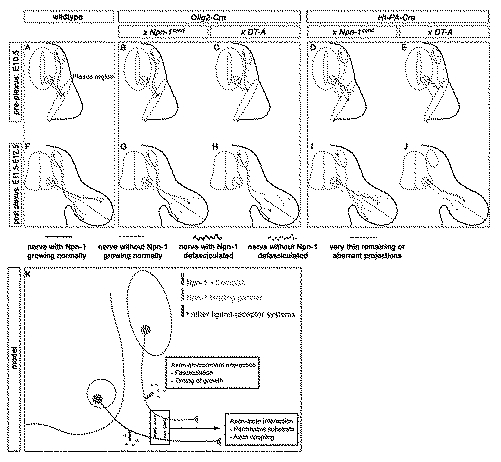
Npn-1-mediated axon-axon and axon-environment interactions. (A) Schematic view of forelimb innervation at E10.5 in wildtype embryos: motor and sensory axons reach the plexus region at the base of the limb in tightly fasciculated spinal nerves. (B) If *Npn-1* is ablated from motoneurons (*Olig2-* or *Hb9-Cre*), motor and sensory axons approach the plexus in normally fasciculated spinal nerves, however motor axons are defasciculated within the plexus. (C) If motoneurons are ablated by tissue-specific activation of the *diphtheria toxin-A* (*DT-A*) gene, only very few, very thin motor projections form at all, while sensory axons grow fasciculated, even though a bit delayed into the plexus region. (D) If Npn-1 is removed from sensory neurons (*Ht-PA-Cre*), sensory and motor axons fail to form properly fasciculated spinal nerves and are therefore defasciculated already before as well as within the plexus region. In addition, sensory neurons lead the spinal nerve projection. (E) If sensory neurons are ablated by tissue-specific DT-A expression, only very thin sensory projections are observed, while motor nerves that grow to the distal limb are defasciculated already before the plexus. (F) Schematic view of forelimb innervation after motor and sensory axons have navigated the plexus region (E11.5–E12.5). (G) If Npn-1 is removed from motoneurons (*Olig2-* or *Hb9-Cre*), motor nerves enter the limb heavily defasciculated, while sensory nerves grow fasciculated to appropriate distal positions. (H) If motor neurons are ablated by tissue-specific activation of *DT-A* (*Olig2-Cre*), sensory axons navigate the decision region of the plexus and show slight alterations in the patterning of their peripheral projections. (I) If Npn-1 is removed from sensory neurons (*Ht-PA-Cre*), both motor and sensory nerves arrive at the plexus in a defasciculated manner, and sensory nerves are heavily defasciculated in the distal limb, while motor projections show a milder defasciculation and grow in slightly inappropriate positions to each other. (J) If sensory neurons are ablated, motor nerves are disorganized and defasciculated before the plexus region as well as in the distal limb, thus showing a similar phenotype to *Npn-1^cond^* ^−*/*−^ *;HT-PA-Cre^+^* mutant embryos. (K) Model illustrating the axon-environment and axon-axon interactions that control the initial outgrowth and joining of sensory and motor axons.

What role do sensory neurons play in the formation of motor projections to the limbs? Removal of Npn-1 specifically from sensory neurons not only had a pronounced effect on the fasciculation of sensory axons but was also associated with defasciculation of motor trajectories (Figure 12). We found no obvious alterations in vasculature and DRG segmentation after *Ht-PA-Cre*-induced removal of Npn-1 (Figure S9). Also, both Schwann cell progenitors and boundary cap cells were clearly present and Schwann cells were found to migrate along defasciculated and normal fiber tracts in similar patterns in mutants and wildtype embryos (Figure S9). The total loss of Schwann cells, such as is the case in *erbB2* deficient mice, has been shown to cause defasciculation of the phrenic nerve in the diaphragm, suggesting that glial cells play a role in axon fasciculation [34]. The largely normal appearance of Schwann cells in *Npn-1^cond^* ^−*/*−^ *;Ht-PA-Cre^+^* mutants makes an effect of Schwann cells appear unlikely, although it cannot be completely excluded at this point. Our data on the defasciculation of motor axons after sensory-specific deletion of Npn-1 were corroborated by genetic elimination of sensory neurons through diphtheria toxin expression, which caused a similar defasciculation of motor fibers prior to the plexus. Particularly interesting is the observation that sensory axons lacking Npn-1 expression are leading motor fibers in their trajectories (Figures 8, 9). This is a surprising result in light of elegant studies showing that in wildtype embryos, at least in the hindlimb later-born sensory axons lag behind motor fibers [5]. A possible explanation may be that Npn-1 presented on sensory fibers produces a tight coupling of sensory to leading motor axons. If Npn-1 is removed from sensory neurons, this “brake” is lifted, sensory axons can overtake motor fibers, and serve as pioneer axons for motor axons to follow. This is in agreement with our observation that sensory axons enter the limb slightly prematurely if Npn-1 is removed from sensory neurons. Complete abolishment of Sema3 binding to Npn-1 in all cells (*Npn-1^Sema^* ^−^) or elimination of *Sema3A* leads to a qualitatively similar though considerably more intense phenotype [10], which might be explained by the residual expression of Npn-1 in sensory neurons in *Npn-1^cond^* ^−*/*−^ *;Ht-PA-Cre^+^* mutants (Figure 1).

Alternatively, the inter-axonal, possibly ligand-independent adhesion emanating from sensory fibers together with Sema3A-mediated surround repulsion may be strong enough to force fasciculation of motor axons with sensory fibers. Upon loss of Npn-1 from sensory fibers, surround repulsion caused by Sema3A may no longer be sufficient to force motor axons into tightly coordinated bundles resulting in defasciculated motor trajectories in spite of Npn-1 still being present in motoneurons. The observation that the motor axon defasciculation phenotype observed upon the removal of Npn-1 from sensory axons is less pronounced than when Npn-1 is ablated from motoneurons supports this explanation (Figure 12). A third explanation might be provided by the neuronal co-expression of Sema3A and Npn-1. It has been shown that Sema3A in motoneurons regulates the level of sensitivity of their growth cones to exogenous Sema3A exposure in the distal limb of the developing chick embryo [35]. This fine-tuning of responsiveness is associated with a local control of the availability of the receptor at the growth cone surface. As Sema3A is also expressed in DRG neurons [29], and sensory and motor axons grow in tight spatial vicinity, it is conceivable that Sema3A secreted from sensory growth cones is not only taken up by sensory but also by motor axons. If Npn-1 is removed from sensory neurons, motor growth cones should be confronted with an excess of Sema3A and in consequence will defasciculate, very similar to the phenotype observed when Sema3A is overexpressed in motoneurons by in ovo electroporation in chick [35]. Aside from class 3 semaphorins, additional, structurally diverse extracellular binding partners have been reported for Npn-1, for example different isoforms of vascular endothelial growth factors [36] or the cell adhesion molecule L1 [37], which may also contribute to the formation of the sensory-motor circuitry.

Intriguingly, the absence of Npn-1 from sensory axons is associated with defasciculation of both motor and sensory axons before, in, and beyond the plexus region in the distal limb. When the cell adhesion molecule L1 was neutralized by injection of function-blocking antibodies into the chick hindlimb at a stage where motor axons have already re-sorted into target-specific bundles, sensory axons chose slightly inappropriate pathways and decreased adhesion was detected ultrastructurally. The pathfinding of motor axons, however, was not affected [38],[39]. From this we conclude that sensory axons affect the fasciculation of motor axons *before* reaching the plexus and experimental defasciculation of sensory projections *after* motor axons have left this decision region has little or no effect on motor axon patterning. Most likely, a concerted effort of both axon-environmental and axon-axonal interactions is required to achieve the remarkable accuracy of limb innervation. While Sema3 ligands constitute the most probable environmental cues to interact with axonally expressed Npn-1, communication between axons might be mediated by Npn-1 homophilic, Npn-1-L1 complexes or even by Sema3A-Npn-1 interactions. A complex combination of molecular mechanisms of axon pathfinding has been reported in other systems including the zebrafish retinotectal projection [28] and MMC motor axon projections [14] and might reflect a general principle in the development of intricate neuronal circuits.

Interestingly, the defasciculation of motor axons that we observed after deletion of Npn-1 in sensory neurons has no effect on the dorsal-ventral pathfinding decision to the limb mesenchyme. This might be due to the less severe degree of defasciculation when compared to the fasciculation defects induced by removal of Npn-1 from motoneurons. In the absence of any markers for dorsally or ventrally projecting sensory neurons, it can currently not be assessed whether sensory axons are still able to correctly navigate this choice point.

Our findings reveal that inter-axonal communication has a pronounced influence on the layout of growth and fasciculation patterns of specific neuronal projections, whereby the spatial region of interaction in relation to important choice points seems to be of critical significance.

## Materials and Methods

### Ethics Statement

Animals were handled and housed according to the federal guidelines for the use and care of laboratory animals, approved by the Helmholtz Zentrum München Institutional Animal Care and Use Committee and the Regierung von Oberbayern.

### Mouse Embryo Preparation

The genotype of mouse embryos was determined as described for *Npn-1^cond^* [17], *Hb9::eGFP* [19], and *DT-A* [23] or using the following primers and conditions to detect the *Cre* allel in *Hb9-Cre* [20], *Ht-PA-Cre* [21], *Isl1-Cre* [22], and *Olig2-Cre* mice [18]: Forward (GTC TCC AAT TTA CTG ACC GTA CAG) and Reverse (GAC GAT GAA GCA TGT TTA GCT GG) primers were used with the following cycling parameters: 5 min preheating to 95°C, 30 cycles of denaturation for 1 min at 95°C, 1 min annealing of the primers at 59.5°C, and 30 s polymerization at 72°C. In all experiments, mutant mice (*Npn-1^cond^* ^−*/*−^ *;Cre^+^*) were compared to control littermates (wt, *Npn-1^cond+/+^;Cre^+^;* or *Npn-1^cond^* ^−*/*−^ *;Cre^wt^*).

### Immunohistochemistry

The protocols for immunohistochemistry and wholemount embryo stainings have been described previously [10]. Immunohistochemical staining against neurofilament in motor nerves was more apparent in younger embryos, where motor nerves appeared yellow due to the overlap in staining for neurofilament and GFP. At E12.5, however, due to the less intense neurofilament labeling, motor nerves appeared green. The following primary antibodies were used for fluorescent immunohistochemistry on cryosections of E10.0–E12.5 embryos or for wholemount embryo preparations of E10.5–E15.5 embryos: Rabbit anti-Lim1 (kindly provided by T.M. Jessell), rabbit anti-GFP (Molecular Probes), rabbit anti-Krox20 (1∶100, Covance, Vertrieb Deutschland: HISS Diagnostics GmbH), rabbit anti-trkA (a generous gift from Lou Reichardt), rabbit anti-Npn-1 (1∶100, a generous gift from Alex Kolodkin), rat anti-PECAM (1∶400, clone Mec13.3, BD Pharmigen), goat anti-TrkC (1∶250, R&D Systems), goat anti-Sox10 (1∶250, Santa Cruz Biotechnology), goat anti-FoxP1 (1∶500, R&D Systems), mouse anti-neurofilament 2H3, and mouse anti-Isl1 39.4D5 (obtained from the Developmental Studies Hybridoma Bank developed under the auspices of the NICHD and maintained by The University of Iowa, Department of Biological Sciences, Iowa City, IA 52242). Antibody staining was visualized using fluorochrome-conjugated secondary antibodies (1∶250; Molecular Probes; Jackson Dianova). For wholemount imaging, embryos were cleared using BABB (1 part benzyl alcohol, 2 parts benzyl benzoate) and imaged using a LSM510 Zeiss confocal microscope. Confocal stacks through the entire extent of the region of interest were acquired and then collapsed for further investigation.

### Quantification of Motor and Sensory Defasciculation at E12.5

To visualize motor defasciculation in wholemount embryos, a perpendicular virtual line of 150 pixel length was placed over a projection picture of confocal planes of the entire limb of Hb9::eGFP-positive nerve branches in fore- and hindlimbs. A plot profile was calculated, resulting in a peak where a gray value above background level crossed that line. To quantify motor defasciculation, the thickness of the four major projections in the forelimb was measured and summarized in control and mutant embryos. In the hindlimb, measurements were performed at the position where tibial and peroneal nerves split up into two branches. Significance was calculated using the two-tailed Student's *t* test. To quantify sensory defasciculation neurofilament positive pixels above background level (without HB9::eGFP) were counted using the imageJ program in a 100×100 pixel area (region of interest, ROI) and significance was calculated using the two-tailed Student's *t* test.

### Quantification of Pre-Plexus Defasciculation at E10.5 and E12.5

To quantify defasciculation before the plexus region in E10.5 and E12.5 wholemount embryos, the individual thickness of the six spinal nerves contributing to the forelimb-plexus was measured (“a” in Figure 8E), added up, and normalized to the length of the spinal cord from which these six projections originate (“b” in Figure 8E) to determine a fasciculation coefficient. Significance was calculated using the two-tailed Student's *t* test.

### Quantification of Distal Advancement

To quantify the distance of ingrowth of motor axons into the fore- and hindlimb of E12.5 embryos, the length of the distal-most motor fiber was measured starting from the reference point and normalized with the length of the forelimb (see Figure 3G for a schematic showing the reference point and the lengths measured). Significance was calculated using the two-tailed Student's *t* test.

### Retrograde Labeling of Neurons

For retrograde labeling of motoneurons, dextran-conjugated Rhodamin (Molecular Probes) in PBS was injected into either dorsal or ventral musculature of E12.5 embryos. Preparations were incubated for 4 h in aerated D-MEM/F12 medium (Gibco) prior to fixation in 4% PFA in PBS and cryoprotection in 30% sucrose in PBS [10]. To quantitate misprojecting neurons, backfilled Rhodamin^+^ neurons were counted, and the percentage of aberrantly projecting neurons was calculated based on immunostaining against Lim1 and Isl1 and significance was calculated using the two-tailed Student's *t* test.

### In Situ Hybridization

In situ hybridization was performed as described [10] using mouse digoxigenin-labeled probes for *Npn-1^cond^*. The fragment of *Npn-1* spanning exon 1 to exon 4 was obtained with the primers 5′-AGGATTTTATGGTTCTTAGG-3′ and 3′-TTGAAGATTTCATAGCGGAT-5′ using Accu Prime Taq (Accu Prime DNA Polymerase System, Invitrogen), cloned into the PCR II topo vector (Topo Cloning Kit, Invitrogen), and antisense probe was created using XhoI (Fermentas) and Sp6 polymerase (Fermentas). To quantify the recombination efficiency of the different Cre-lines, in situ hybridization against the floxed exon 2 of Npn-1 was performed on mutant and wildtype littermate embryos. For *Npn-1^cond^* ^−*/*−^ *;Olig2-Cre^+^* embryos the percentage of Npn-1^+^/Isl-1^+^ cells was calculated for the medial LMC (Isl1^+^ cells), and the percentage of Npn-1^+^/Lim-1^+^ cells was calculated for the lateral LMC (Lim1^+^ cells). To quantify the recombination efficiency of the *Ht-PA-Cre* line in sensory neurons, in situ hybridization against the floxed exon 2 of Npn-1 was performed and the percentage of Npn-1^+^/Isl-1^+^ cells per DRG (Isl1^+^ cells) was calculated. Significance was calculated using the two-tailed Student's *t* test.

## Supporting Information

Figure S1 Dorsal view of brachial plexus and spinal nerve exit points. Dorsal view of the plexus region and exit of brachial spinal nerves from the spinal cord at E12.5. N. musculocutaneous (msc), n. radialis (rad), n. ulnaris (uln), and n. cutaneous maximus (cm) can be identified in control embryos at E12.5 (A). Spinal nerves are fasciculated from their exit of the spinal cord until they reach the plexus in *Npn-1^cond^* ^−*/*−^ *;Olig2-Cre^+^* mutant embryos (B, F), however, after the plexus motor nerves are thinner, defasciculated (e.g. radial nerve), or stunted (e.g. ulnar nerve). Defasciculation after the plexus was also observed in *Npn-1^cond^* ^−*/*−^ *;Hb9-Cre^+^* mutant embryos (C), while the spinal nerves were fasciculated from the spinal cord (sc) to the plexus (G, I). In *Npn-1^cond^* ^−*/*−^ *;Ht-PA-Cre^+^* mutant embryos spinal nerves arrived at the plexus in a slightly defasciculated manner (H). Motor nerves were also disorganized after the plexus region, particularly the ulnar nerve (uln) and also a smaller branch of the radial nerve (empty arrowhead) seem to consist of more nerve branches than in control animals. Quantification of the pre-plexus defasciculation was carried out as described for E10.5 embryos in Figure 8; both sides of the spinal cord were analyzed for 3 embryos; p^Olig2-Cre^ = 0.92, p^Hb9-Cre^ = 0.84, p^Ht-PA-Cre^ < 0.01. Bar graph equals 200 µm for (A–D) and 100 µm for (E–H).
(EPS)

Figure S2 Defasciculation of motor projections after *Hb9-Cre*-mediated removal of Npn-1 from motoneurons. Wholemount antibody staining of E12.5 embryos against GFP (green, motor nerves) and neurofilament (red, motor and sensory nerves). Ablation of *Npn-1* from motoneurons using the *Hb9-Cre* line leads to defasciculation of the radial and median nerves (B, D, *n* = 6), but not the ulnar nerve (arrow in D) when compared to wildtype littermates (A, C). The fasciculation and distal positioning of sensory nerves is not affected by defasciculation of motor trajectories (arrowheads in B') when compared to littermate controls (arrowheads in A'). High magnification of the ulnar nerve shows normal development of motor and sensory components in *Npn-1^cond^* ^−*/*−^ *;Hb9-Cre^+^* mutants (F, F') compared to controls (E, E'). Bar equals 400 µm in (A, B), 100 µm in (C, F), and 80 µm in (E, F).
(EPS)

Figure S3 Removal of *Npn-1* from motor or sensory neurons causes severe defasciculation of motor axons in the hindlimb. Analysis of GFP immunofluorescence in wholemount E12.5 embryos reveals that motor axons of the sciatic nerve (peroneal branch) are severely defasciculated and fanned out in *Npn-1^cond^* ^−*/*−^ *;Olig2-Cre^+^* (C, D, *n* = 10) and *Npn-1^cond^* ^−*/*−^ *;Hb9-Cre^+^* (E, F, *n* = 6) mutant embryos instead of forming distinct nerve trunks as in controls (A, B). Interestingly, motor projections are also defasciculated when Npn-1 is removed from sensory neurons using the *Ht-PA-Cre* line (G, H, *n* = 5), in particular the tibial nerve (arrow in H). Here, we also observe loop formation of the superficial branch of the peroneal nerve (H, open arrowhead). Wholemount antibody staining against neurofilament shows that defasciculation of motor projections and lack of major rami does not affect fasciculation and distal positioning (arrowheads in B', D', F') of sensory nerves to the hindlimb in *Olig2-Cre* (C', D') or *Hb9-Cre* (E', F') mutant embryos when compared to littermate controls (A', B'). Elimination of Npn-1 from sensory neurons causes a strong defasciculation and exuberant growth in *Npn-1^cond^* ^−*/*−^ *;Ht-PA-Cre^+^* mutant animals (G', H'). Bar equals 400 µm in (A, C, E, G) and 100 µm in (B, D, F, H).
(EPS)

Figure S4 Fasciculation, but not distal advancement, of motor and sensory projections is affected after ablation of *Npn-1* from motor and sensory neurons in the hindlimb. Defasciculation of motor nerves was assessed by calculating a plot profile of Hb9::eGFP positive motor projections crossing a virtual line. In control embryos (A and B) the two major projections of the sciatic nerve can be seen (1 = n. peroneous, 2 = n. tibialis). In *Npn-1^cond^* ^−*/*−^ *;Olig2-Cre^+^* mutant embryos (C and D) the tibial nerve (2) can be assigned to the plot profile, even though smaller fibers are defasciculating from the main branch. The peroneal nerve (1) is split up into many small projections that did not merge to a fascicle when growing into the distal limb. This is also observed in *Npn-1^cond^* ^−*/*−^ *;Hb9-Cre^+^* mutant embryos (E and F), with the peroneal nerve (1) showing many small fibers that are separated from the main nerve trunk, whereas the tibial nerve (2) appears normal. Ablation of *Npn-1* from sensory neurons by *Ht-PA-Cre* (G and H) causes defasciculation of motor projections, shown in the plot profile to result particularly in defasciculation of the tibial nerve (2). The quantification of the defasciculation by summarizing the thicknesses of tibial and peroneal nerves (I) revealed increased defasciculation in *Npn-1^cond^* ^−*/*−^ *;Olig2-Cre^+^* (62.3±5.5 SEM, *p*<0.0001), *Npn-1^cond^* ^−*/*−^ *;Hb9-Cre^+^* (100.1±9.7 SEM, *p*<0.0001), and *Npn-1^cond^* ^−*/*−^ *;Ht-PA-Cre^+^* (41.1±4.9 SEM, *p*<0.005) mutants when compared to wildtype littermates (17.7±0.8 SEM). Note that the higher thickness of hindlimb nerves in *Npn-1^cond^* ^−*/*−^ *;Hb9-Cre^+^* does not indicate a more severe degree of fasciculation, but rather a wider spread of defasciculated nerve branches. The distal advancement in the hindlimb was quantified by measuring the length of the most distal axon and normalizing this to the length of the hindlimb and was found unaltered if Npn-1 is removed from sensory or motor neurons (J). Also the distal advancement of motor innervation in forelimbs of *Npn-1^cond^* ^−*/*−^ *;Ht-PA-Cre^+^* mutant embryos was unaffected (J). Quantification of sensory defasciculation in the hindlimb does not show differences in *Npn-1^cond^* ^−*/*−^ *;Olig2-Cre^+^* (L) and *Npn-1^cond^* ^−*/*−^ *;Hb9-Cre^+^* (M) mutant embryos when compared to littermate controls (K). The quantification in (O) shows a significant increase of defasciculated sensory innervation in *Npn-1^cond^* ^−*/*−^ *;Ht-PA-Cre^+^* mutant embryos (N). *n* = 3 for all genotypes; both limbs were quantified. Bar graph in (N) equals 100 µm for all panels.
(EPS)

Figure S5 Defasciculation of MMC projections after removal of Npn-1. Wholemount antibody staining of E12.5 embryos against GFP (green, motor nerves) and neurofilament (red, motor and sensory nerves). Elimination of *Npn-1* from motoneurons using the *Olig2-Cre* line leads to misprojections of MMC nerve branches innervating intercostal muscles with axons crossing between the main nerve bundles (C, arrowheads in D), a behavior that was only very rarely observed in control embryos (A, B, E; *p* = 0.0052, *n* = 6). Ablation of *Npn-1* from sensory neurons by *Ht-PA-Cre* leads to defasciculation of sensory (G') and motor (G) projections at thoracic levels. Motor axons cross frequently between major nerve bundles (arrowheads in G, E; *p*<0.0001, *n* = 4). Bar equals 500 µm in (A, C, F) and 200 µm in (B, D, G).
(EPS)

Figure S6 Defasciculation of intercostal axons after removal of Npn-1 at E15.5. Wholemount immunohistochemistry for neurofilament (red, motor and sensory nerves) and GFP (green, motor nerves). Removal of *Npn-1* from motoneurons causes defasciculation of intercostal nerves in *Npn-1^cond^* ^−*/*−^;*Olig2-Cre^+^* mutants with axon bundles frequently de- and refasciculating with the main nerve (arrows in B, C). Bar equals 200 µm in all panels.
(EPS)

Figure S7 Npn-1 is expressed in TrkC-positive sensory neurons at E12.5. Anti-TrkC staining (A) and in situ hybridization against *Npn-1* (B) on the same section demonstrate that 19.0%±1.9% SEM of TrkC-positive sensory neurons at brachial and 16.5%±0.8% SEM at lumbar spinal levels also express *Npn-1* at E12.5. Scale bar equals 25 µm in all panels.
(EPS)

Figure S8 No dorsal-ventral guidance defects in sensory neuron-specific ablation of Npn-1. Retrograde tracing from ventral (A) and dorsal (B) limb mesenchyme of *Npn-1^cond^* ^−*/*−^;*Ht-PA-Cre^+^* mutant embryos did not show an increase of pathfinding errors at brachial (2.95%±0.59% and 3.86%±0.84%, respectively, *n* = 3) nor lumbar levels (5.1%±0.82% and 4.33%±1.31%, respectively, *n* = 3) when compared to wildtype littermate embryos (forelimb^ventral^ = 4.99%±0.76%; forelimb^dorsal^ = 3.56%±0.47%; hindlimb^ventral^ = 5.19%±0.77%; hindlimb^dorsal^ = 4.21%±0.84%).
(EPS)

Figure S9 Assessment of DRG segmentation, Schwann cell progenitors, and boundary cap cell formation as well as blood vessel formation in *Npn-1^cond^* ^−*/*−^ *;Ht-PA-Cre^+^* mutant embryos. Dorsal view of the spinal cord and DRG of E12.5 wholemount embryos revealed that the segmentation of DRG is normal in *Npn-1^cond^* ^−*/*−^ *;Ht-PA-Cre^+^* mutant embryos (B); in particular, no fusions or aberrant morphology were observed when compared to littermate controls (A). Schwann cell progenitor formation was assessed by SOX10 immunohistochemistry, showing SOX10^+^ progenitor cells following a defasciculated distal nerve branch in the forelimb and in DRG (inlay in D and C) of mutant embryos (D). A control embryo is shown in (C). Also the formation of boundary cap cells (KROX20^+^) at the dorsal root entry zone (DREZ) and motor entry zone (MEZ) is not impaired in *Npn-1^cond^* ^−*/*−^ *;Ht-PA-Cre^+^* mutant embryos (F, control embryo is shown in E). The formation of blood vessels was assessed by staining with anti-PECAM antibody and revealed no obvious differences between mutant (G) and control (H) embryos. Bar graph equals 100 µm for (A, B), 40 µm for (C, D), 80 µm for inlays in (C, D), 20 µm for (E, F), and 150 µm for (G, H).
(EPS)

## Abbreviations

DREZ: dorsal root entry zone
DRG: dorsal root ganglia
FL: forelimb
HL: hindlimb
LMC: lateral motor column
MEZ: motor entry zone
MMC: medial motor column
Npn-1: Neuropilin-1
Sema3: Semaphorin class 3
